# Supplementary material for: Implementation of Clinical Practice Guidelines for Empirical Antibiotic Therapy of Bacteremia, Urinary Tract Infection, and Pneumonia: A Multi-Center Quasi-Experimental Study
Source: Antibiotics (Basel). 2022 Jul 6;11(7):903. doi: 10.3390/antibiotics11070903 (PMC9311984; doi:10.3390/antibiotics11070903)
Supplement: Supplementary file 1 [file antibiotics-11-00903-s001.zip › antibiotics-1791266-supplementary.pdf]

Supplementary Materials

Table S1. Clinical Practice Guidelines, Sakaeo Crown Prince Hospital.

**Targeted Infection**	**Types of infection and MDR risk**	**Recommended empirical ATB**	**Notes**
Bacteraemia	CAI, without MDR risk	Ceftriaxone	-If rickettsial infection is suspected: add doxycycline or azithromycin<br>-If *B. pseudomallei* is suspected: replace ceftriaxone with ceftazidime
	CAI, with MDR risk	Amoxicillin/clavulanate<br>or<br>Piperacillin/tazobactam	-If rickettsial infection is suspected: add doxycycline or azithromycin<br>-If *B. pseudomallei* is suspected: replace ceftriaxone with ceftazidime
	HAI	Piperacillin/tazobactam<br>or<br>Meropenem	-If MRSA is suspected: add vancomycin
	HAI while receiving carbapenem	Colistin	-If MRSA is suspected: add vancomycin
**Targeted Infection**	**Types of infection and MDR risk**	**Recommended empirical ATB**	**Notes**
Pneumonia	CAI, without MDR risk	Ceftriaxone and azithromycin	-If aspiration pneumonia is suspected: replace ceftriaxone with Amoxicillin/clavulanate<br>-If influenza is suspected: add oseltamivir<br>-If *B. pseudomallei* is suspected: replace ceftriaxone with ceftazidime
	CAI, with MDR risk	Ceftriaxone and azithromycin<br>or<br>Levofloxacin monotherapy	-If influenza is suspected: add oseltamivir<br>-If *B. pseudomallei* is suspected: replace ceftriaxone with ceftazidime
	HAI	Piperacillin/tazobactam<br>or<br>Meropenem	-If MRSA is suspected: add vancomycin
	HAI with suspected carbapenem-resistant gram-negative organisms	Colistin	-If MRSA is suspected: add vancomycin
**Targeted Infection**	**Types of infection and MDR risk**	**Recommended empirical ATB**	**Notes**
UTI	CAI, without MDR risk	Ceftriaxone	-If mild clinical syndrome, no shock or hypotension: oral cephalosporin could be used

	CAI, with MDR risk	-Amikacin<br>or<br>-IV Amoxicillin/clavulanate<br><br>-Piperacillin/tazobactam or Ertapenem	-Normal renal function<br><br>-Impaired renal function<br><br>-If 3rd generation cephalosporin resistant is suspected
	HAI	Piperacillin/tazobactam<br>or Ertapenem<br>or Meropenem	-If MRSA or *Enterococcus faecium* is suspected: add vancomycin
	HAI with suspected carbapenem-resistant gram-negative organisms	Colistin	-If MRSA or *Enterococcus faecium* is suspected: add vancomycin

Table S2. Clinical Practice Guidelines, Surin Hospital.

Targeted Infection	Types of infection and MDR risk	Recommended empirical ATB	Notes
Bacteraemia	CAI, without MDR risk	Ceftriaxone	-If rickettsial infection is suspected: add doxycycline or azithromycin<br>-If *B. pseudomallei* is suspected: replace ceftriaxone with ceftazidime
	CAI, with MDR risk	Amoxicillin/clavulanate<br>or<br>Piperacillin/tazobactam	-If rickettsial infection is suspected: add doxycycline or azithromycin<br>-If *B. pseudomallei* is suspected: replace ceftriaxone with ceftazidime
	HAI	Piperacillin/tazobactam<br>or<br>Meropenem	-If MRSA is suspected: add vancomycin
	HAI while receiving carbapenem	Colistin	-If MRSA is suspected: add vancomycin
**Targeted Infection**	**Types of infection and MDR risk**	**Recommended empirical ATB**	**Notes**
Pneumonia	CAI, without MDR risk	Ceftriaxone and azithromycin	-If aspiration pneumonia is suspected: replace ceftriaxone with Amoxicillin/clavulanate<br>-If influenza is suspected: add oseltamivir<br>-If *B. pseudomallei* is suspected: replace ceftriaxone with ceftazidime
	CAI, with MDR risk	Ceftriaxone and azithromycin<br>or<br>Levofloxacin monotherapy	-If influenza is suspected: add oseltamivir<br>-If *B. pseudomallei* is suspected: replace ceftriaxone with ceftazidime
	HAI	Piperacillin/tazobactam<br>or<br>Meropenem	-If MRSA is suspected: add vancomycin
	HAI with suspected carbapenem-resistant gram-negative organisms	Colistin	-If MRSA is suspected: add vancomycin
**Targeted Infection**	**Types of infection and MDR risk**	**Recommended empirical ATB**	**Notes**
UTI	CAI, without MDR risk	Ceftriaxone	-If mild clinical, no shock or hypotension: oral cephalosporin could be used
	CAI, with MDR risk	-Amikacin<br>-IV Amoxicillin/clavulanate<br>-Piperacillin/tazobactam	-Normal renal function<br>-Abnormal renal function<br><br>-If 3rd generation

		or Ertapenem	cephalosporin resistant is suspected and normal renal function
	HAI	Piperacillin/tazobactam<br>or Ertapenem<br>or Meropenem	-If MRSA or *Enterococcus faecium* is suspected: add vancomycin
	HAI with suspected carbapenem-resistant gram-negative organisms	Colistin	-If MRSA or *Enterococcus faecium* is suspected: add vancomycin

Table S3. Clinical Practice Guidelines, Surat Thani Hospital.

**Targeted Infection**	**Types of infection and MDR risk**	**Recommended empirical ATB**	**Notes**
Bacteraemia	CAI, without MDR risk	Ceftriaxone	-If rickettsial infection is suspected: add doxycycline or azithromycin
	CAI, with MDR risk	Amoxicillin/clavulanate or Piperacillin/tazobactam	-If rickettsial infection is suspected: add doxycycline or azithromycin
	HAI	Piperacillin/tazobactam or Meropenem	-If MRSA is suspected: add vancomycin
	HAI while receiving carbapenem	Colistin	-If MRSA is suspected: add vancomycin
**Targeted Infection**	**Types of infection and MDR risk**	**Recommended empirical ATB**	**Notes**
Pneumonia	CAI, without MDR risk	Ceftriaxone and azithromycin	-If aspiration pneumonia is suspected: replace ceftriaxone with Amoxicillin/clavulanate<br>-If influenza is suspected: add oseltamivir
	CAI, with MDR risk	Ceftriaxone and azithromycin or Levofloxacin monotherapy	-If influenza is suspected: add oseltamivir
	HAI	Piperacillin/tazobactam or Meropenem	-If MRSA is suspected: add vancomycin
	HAI with suspected carbapenem-resistant gram-negative organisms	Colistin	-If MRSA is suspected: add vancomycin
**Targeted Infection**	**Types of infection and MDR risk**	**Recommended empirical ATB**	**Notes**
UTI	CAI, without MDR risk	Ceftriaxone	-If mild clinical, no shock or hypotension: oral cephalosporin could be used
	CAI, with MDR risk	-Amikacin<br>-IV Amoxicillin/clavulanate<br>-Piperacillin/tazobactam or Ertapenem	-Normal renal function<br>-Abnormal renal function<br><br>-If 3rd generation cephalosporin resistant is suspected and normal renal function
	HAI	Piperacillin/tazobactam or Ertapenem or Meropenem	-If MRSA or Enterococcus faecium is suspected: add vancomycin
	HAI with suspected carbapenem-resistant gram-negative organisms	Colistin	-If MRSA or Enterococcus faecium is suspected: add vancomycin

Abbreviations and definitions for CPGs.

- CAIs, community-acquired infections: onset of symptoms occurs before hospitalisation/within 48 hours after hospitalisation and there are no healthcare associated conditions potentially related to infection (i.e., chronic haemodialysis, in-dwelling urinary catheter).
- HAIs, hospital-acquired infections: onset of symptoms occurs >48 hours after hospitalisation or there are healthcare associated conditions potentially related o infection (i.e., chronic haemodialysis, in-dwelling urinary catheter).
- MDR, multidrug resistant.
- MRSA, methicillin-resistant *Staphylococcus aureus.*

Risk factors for infection by MDR pathogens.

- Hospitalisation within 90 days
- Antimicrobials use within 90 days
- Previous infection with antimicrobial resistant organisms
- Treatment in intensive care unit settings for longer than 48 hours
- Renal replacement therapy
- Medical device insertion or ventilator use longer than 48 hours
